# Suppression of Reactive Oxygen Species Accumulation in Chloroplasts Prevents Leaf Damage but Not Growth Arrest in Salt-Stressed Tobacco Plants

**DOI:** 10.1371/journal.pone.0159588

**Published:** 2016-07-21

**Authors:** Anabella F. Lodeyro, Mariana Giró, Hugo O. Poli, Gabriel Bettucci, Adriana Cortadi, Alejandro M. Ferri, Néstor Carrillo

**Affiliations:** 1 Instituto de Biología Molecular y Celular de Rosario (IBR-UNR/CONICET), Facultad de Ciencias Bioquímicas y Farmacéuticas, Universidad Nacional de Rosario (UNR), 2000, Rosario, Argentina; 2 Department of Biological Sciences, Facultad de Ciencias Bioquímicas y Farmacéuticas, Universidad Nacional de Rosario (UNR), 2000, Rosario, Argentina; 3 Department of Analytical Chemistry, Facultad de Ciencias Bioquímicas y Farmacéuticas, Universidad Nacional de Rosario (UNR), 2000, Rosario, Argentina; The Chinese University of Hong Kong, HONG KONG

## Abstract

Crop yield reduction due to salinity is a growing agronomical concern in many regions. Increased production of reactive oxygen species (ROS) in plant cells accompanies many abiotic stresses including salinity, acting as toxic and signaling molecules during plant stress responses. While ROS are generated in various cellular compartments, chloroplasts represent a main source in the light, and plastid ROS synthesis and/or elimination have been manipulated to improve stress tolerance. Transgenic tobacco plants expressing a plastid-targeted cyanobacterial flavodoxin, a flavoprotein that prevents ROS accumulation specifically in chloroplasts, displayed increased tolerance to many environmental stresses, including drought, excess irradiation, extreme temperatures and iron starvation. Surprisingly, flavodoxin expression failed to protect transgenic plants against NaCl toxicity. However, when high salt was directly applied to leaf discs, flavodoxin did increase tolerance, as reflected by preservation of chlorophylls, carotenoids and photosynthetic activities. Flavodoxin decreased salt-dependent ROS accumulation in leaf tissue from discs and whole plants, but this decline did not improve tolerance at the whole plant level. NaCl accumulation in roots, as well as increased osmotic pressure and salt-induced root damage, were not prevented by flavodoxin expression. The results indicate that ROS formed in chloroplasts have a marginal effect on plant responses during salt stress, and that sensitive targets are present in roots which are not protected by flavodoxin.

## Introduction

Salinity is one of the major abiotic stresses affecting plant productivity worldwide. While virtually all salts may have negative effects on plant growth and reproduction, the term salt stress usually refers to the damage caused by NaCl, since this is by far the most prevailing environmental stress related to salinity [1, 2]. NaCl exerts its toxic effects at various levels. In a short-scale time frame, minutes to hours after salt exposure, plants undergo osmotic stress which results in water limitation, stomatal closure and growth arrest of roots and shoots. Subsequently, Na^+^ and Cl^-^ may enter the cell where they can inhibit key enzymes and metabolic pathways (ionic stress), and cause disturbances of the ion transport systems resulting in K^+^ and Ca^2+^ limitation (nutritional stress) [3–5]. Finally, salt-dependent inhibition of gas exchange and photosynthesis may lead to runaway production of reactive oxygen species (ROS) and the establishment of an oxidative stress condition [6–8]. Osmotic, nutritional, ionic and oxidative stress, all can contribute to the damage experienced by plants exposed to excess NaCl. Several of these symptoms are shared with other abiotic stresses, especially drought and cold. Plants respond to the adverse effects of salinity through multiple protective mechanisms, including control of water loss, osmotic and metabolic adjustment via the synthesis of compatible osmolytes, active extrusion of the toxic ions and ROS detoxification [1, 4, 7, 9]. These responses involve a massive reprogramming in the expression of the plant genome [10]. Unlike halobacteria, salt tolerance in plants relies on cellular and biochemical mechanisms of salt avoidance rather than on the evolution of salt-resistant proteins and enzymes (reviewed in [11]).

Increased ROS production is a common feature of many different conditions of abiotic stress besides salinity [7, 12–14]. ROS are reported to act both as toxic molecules accounting for a significant fraction of the damage undergone by the stressed plants, and as signaling cues to trigger specific protective responses [15, 16]. ROS can be synthesized in various sub-cellular compartments by different pathways. Early plant responses to saline environments include reduction of stomatal conductance to avoid water loss [6], which in turn decreases internal CO_2_ concentrations and slows down carbon assimilation by the Calvin cycle. As the regenerative step of the cycle ceases to use NADPH under these conditions, the immediate consequence is NADP^+^ depletion and delivery of the excess of energy and reducing equivalents to O_2_, with concomitant formation of ^1^O_2_, O_2_^.-^ and H_2_O_2_ in chloroplasts [17]. In addition, the decline in the carboxylase reaction of Rubisco stimulates photorespiration in C3 plants, leading to H_2_O_2_ generation in peroxisomes [18]. Salinity also increases respiratory rates, with the consequent electron leakage to O_2_ in mitochondria [7]. Finally, the plasma membrane-bound NADPH oxidase and the apoplastic diamine oxidase are activated during salt stress and therefore contribute to extracellular ROS propagation [15, 19].

The specific contribution of chloroplast-generated ROS to the damage and tolerance of plants exposed to abiotic stress episodes has been studied using a variety of approaches, most conspicuously modification of the plastidic ROS scavenging systems by mutation and/or overexpression of specific components [2, 20, 21]. Attempts to improve stress tolerance by manipulation of these antioxidant systems showed variable degrees of success, reflecting the intricacies of the multigenic plant responses [22], and the difficulties of identifying potential intervention points that confer tolerance to multiple stresses and at the same time do not introduce penalties to plant growth and/or development [23].

An alternative approach that showed great potential was based on the use of the cyanobacterial protein flavodoxin (Fld), an electron transfer shuttle involved in stress protection in prokaryotes and marine algae, but absent from the plant genome [24]. Flds contain flavin mononucleotide as prosthetic group and participate in many oxido-reductive pathways including photosynthesis, in which they can act as a replacement of the isofunctional iron-sulfur protein ferredoxin (Fd) [25, 26]. Flds are usually induced under conditions of environmental stress and iron starvation, when Fd levels are down-regulated, allowing survival and reproduction of the microorganism under these adverse conditions [24, 25, 27]. As indicated before, Fld is not found in plants but introduction of an engineered cyanobacterial gene encoding a plastid-targeted Fld into the nuclear genome of various model and crop species led to stable transformants that displayed enhanced tolerance, at the whole plant level, to multiple sources of environmental stress, including drought, extreme temperatures, redox-cycling herbicides, high light, UV radiation and iron deficit [28–30]. By productively interacting with chloroplast redox partners, Fld was able to drive reducing equivalents away from oxygen and deliver them into metabolic, dissipative and regulatory pathways of the plastid, therefore preventing ROS formation [28, 31, 32]. In this sense, Fld does not behave as a typical scavenger reacting with one or various ROS and rendering them harmless, but acts instead as a plastid-specific general antioxidant by avoiding propagation of the different ROS species formed under stress as a consequence of excess excitation energy on the photosynthetic electron transport chain [24].

These observations lent support to the notion that chloroplast-borne ROS could play a critical role during plant responses to adverse environments, and since ROS build-up is a significant component of salt stress [7, 12, 14], it was expected that Fld expression could also alleviate the adverse effects of salinity. However, *Medicago* plants expressing Fld in chloroplasts exhibited wild-type (WT) levels of tolerance against NaCl toxicity [33]. This result was somehow surprising since significant contribution of Fld to protection against salt stress had been reported in several cyanobacterial species [34, 35], suggesting that the salinity syndrome presents some particular aspects in plants that are not shared by other photosynthetic organisms or environmental stresses.

The purpose of the present research was therefore to investigate this apparent paradox and to characterize the salt response of transgenic tobacco plants expressing Fld. Different salt stress treatments were applied to various tissues, from leaf discs to whole plants, and the responses of WT and Fld-expressing plants were evaluated with respect to growth, tissue damage, viability, and salt and ROS accumulation. While Fld decreased salt-dependent ROS build-up in both leaf discs and attached leaves, this decline failed to improve the salt tolerance of whole plants. Indeed, growth inhibition, leaf pigment degradation and root damage were similar in WT and *pfld* plants exposed to NaCl. The results indicate that the oxidative component of salt stress is largely confined to photosynthetic tissues, while other targets, presumably located in the roots, are responsible for the salt-dependent growth arrest of the stressed plants, suggesting that control of chloroplast-generated ROS is not an attractive intervention point for the design of salt-tolerant crops.

## Materials and Methods

### Plant material and growth conditions

Preparation and characterization of tobacco plants (*Nicotiana tabacum* cv. Petit Havana) constitutively expressing a plastid-targeted *Anabaena* PCC7119 Fld under the control of the cauliflower mosaic virus (CaMV) 35S promoter (*pfld* lines) have been described elsewhere [28]. Homozygous lines *pfld*5-8 and *pfld*4-2, used throughout this research, accumulate 70 and 57 pmol of Fld per g of leaf fresh weight (FW), respectively, in the same range of endogenous Fd [28].

WT and *pfl*d seeds were germinated on Murashige-Skoog (MS0)-agar plates [36] supplemented with 3% (w/v) sucrose and, in the case of transformants, 100 μg mL^-1^ kanamycin. After 4 weeks, seedlings were transferred to soil or grown hydroponically in Hoagland nutrient medium (HM) [37], in both cases in controlled environment chambers under a 16-h photoperiod, with an irradiance of 250 μmol m^-2^ s^-1^, a relative humidity of 60% and day/night temperatures of 28/23°C (growth chamber conditions).

### Stress treatments

All stress experiments were carried out under growth chamber conditions. The effect of NaCl on whole plants was evaluated in MS0-agar, or in HM hydroponic medium. In the first case, seedlings were grown for 12–15 days, until the first two leaves were fully expanded. Seedlings were then transferred to new MS0-agar plates containing different concentrations of NaCl, and cultured for additional 11–15 days. Osmotic pressures of the NaCl mixtures were calculated using the van’t Hoff formalism with a correction coefficient of 1.8 [38]. For osmotic treatments, MS0-agar plates were infused with polyethylenglycol (PEG 8000) as described by Verslues et al. [39].

For experiments in hydroponics, plants were grown in HM for 4 weeks before transfer to fresh medium supplemented with NaCl.

To evaluate the effects of salt stress directly in leaf tissue, discs (1.2 cm in diameter) were punched from the fourth fully expanded leaf of 8-week-old plants grown in soil, floated with the abaxial side down and incubated with distilled water or the NaCl solution.

### Microscopic analysis of root tissues

For microscopic observations, root samples were fixed for 2 h at 4°C in 0.1 M sodium cacodylate, pH 7.2, containing 4% (v/v) glutaraldehyde, washed for 20 min in cacodylate buffer supplemented with 10% (w/v) sucrose, and dehydrated by successive passages (1 h each) through a graded series of *n*-butanol solutions of increasing concentrations (10%, 20%, 35% and 50%) in 50% (v/v) ethanol, followed by final passages in 75% and 100% *n*-butanol. Samples were finally embedded in paraffin for the preparation of permanent slides using xylol as solvent. A double staining procedure with safranin and Fast Green was used to visualize root tissues. Cross sections (8–10 μm width) were incubated for 30 min at 25°C with a saturated solution of safranin in 80% (v/v) ethanol, washed with ethanol for 1 min, stained for 30 s with a saturated solution of Fast Green in ethanol and washed twice with ethanol, followed by a final incubation (5 min) with xylol. Sections were mounted using Canada balsam, and micrographs taken with an Olympus CH30 microscope equipped with a digital camera (Samsung NX11).

### Determination of photosynthetic parameters

Measurements of chlorophyll fluorescence associated to photosystem II (PSII) were carried out at 25°C on dark-adapted leaves using a Qubit Systems pulse-modulated fluorometer. The *Fv* and *Fm* parameters were determined after 30 min in the dark, and the light-adapted values (*Fv’* and *Fm’*) were measured after 30 min of illumination at 200 μmol m^-2^ s^-1^. Photosynthetic parameters *Fv*/*Fm*, Φ_PSII_, non-photochemical quenching (NPQ) and *q*P were calculated as described [40].

### ROS measurements and localization

Lipid hydroperoxides (LOOH) were measured in cleared leaf and root extracts using a modified ferrous oxidation-xylenol orange assay as described by DeLong et al. [41], using the reducing agent triphenylphospine to discriminate LOOH from interfering compounds. LOOH contents in the various extracts were quantified by comparison with a H_2_O_2_ calibration curve [41].

ROS cellular localization was determined by confocal microscopy using the fluorescent probe 2´, 7´-dichlorofluorescein diacetate (DCFDA). Leaf tissue was vacuum-infiltrated in the dark with 50 μM DCFDA in 10 mM Tris-HCl pH 7.5, and ROS were visualized in an Eclipse TE– 2000 –E2 Nikon confocal microscope with excitation at 488 nm and emission at 515/530 nm. Fluorescence intensities were quantified using the Image J software.

### Determination of Na^+^ and K^+^ contents

For measurements of Na^+^ and K^+^ in plant tissues, leaves and roots from treated and control plants and leaf discs were collected, rinsed with distilled water and dried to constant dry weight (DW) at 60–80°C during 24–48 h in a forced-air oven. Samples were then ground into fine powder with a mortar and pestle and extracted with concentrated HNO_3_ at 25°C for 24 h followed by 1 h of boiling. Subsequently, H_2_O_2_ was added to 30% (v/v) and boiled for an additional hour. Na^+^ and K^+^ contents in the solutions were determined by atomic absorption spectroscopy using a Unicam 969 spectrometer.

### Osmotic measurements

To estimate the osmolarity of root extracts, roots were homogenized with 0.1 mL of distilled water and centrifuged at 10,000 x *g* for 5 min. The supernatants were collected and their osmotic pressures were measured using with a Gonotec Osmomat 030 cryoscopic osmometer [42]. The osmotic potential of whole roots was determined in C-52 Wescor sample chambers coupled to a Dew Point Microvoltimeter HR-33T [43]. Tissue corresponding to 3 cm of roots from plants grown on MS0-agar plates was used in each chamber.

### Analytical procedures

Chlorophylls and carotenoids were determined spectrophotometrically after extraction from leaves and leaf discs with 96% (v/v) ethanol [44].

Root and leaf viabilities were determined by measuring reduction of the dehydrogenase substrate 2,3,5-triphenyltetrazolium chloride (TTC) in cleared extracts, as described by Steponkus and Lanphear [45]. Viabilities were expressed as the absorbance change per g FW.

### Statistical analyses

Data were usually analyzed using two-way ANOVA and Holm-Sidak multiple range tests. When the normality and/or equal variance assumptions were not met, Kruskal–Wallis one-way ANOVA between lines within each NaCl treatment, and Tukey multiple range tests were used. In all cases, significant differences refer to statistical significance at P ≤ 0.05. The specific statistics applied in each experiment are indicated in the corresponding figure legends.

## Results

### Flavodoxin expression protects tobacco leaf discs, but not whole plants, from salt toxicity

**Fig 1:**
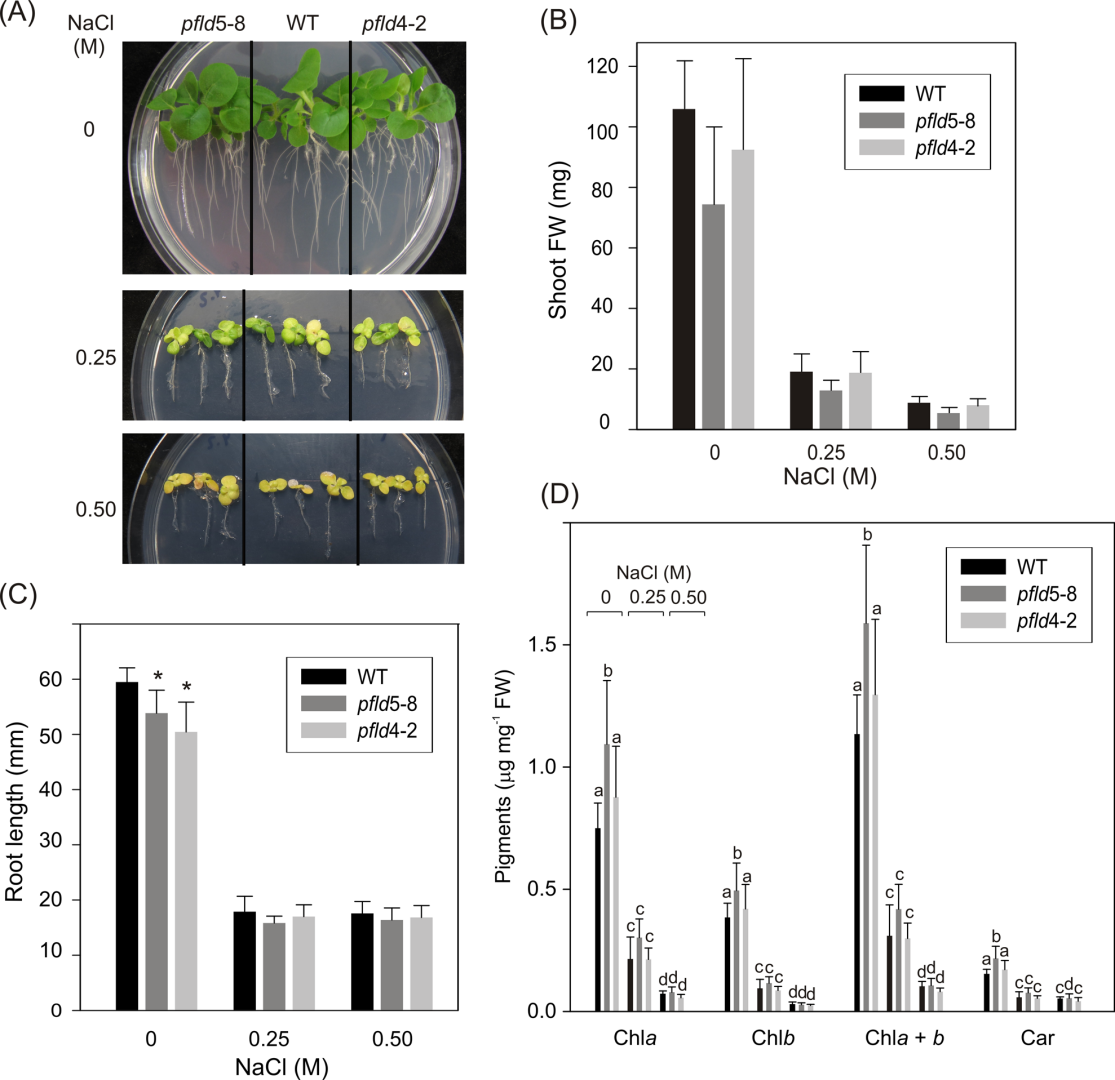
Flavodoxin expression in chloroplasts does not increase tolerance of tobacco plants to salinity. Twelve-day old WT and *pfld* seedlings cultured in MS0-agar plates were incubated with NaCl in the same medium and photographed after 15 days of treatment (A). Fresh weight (FW) of the aerial parts (B), and root length (C) were determined in 12 independent specimens. Asterisks in (C) indicate significant differences of the means between lines in the absence of NaCl at P ≤ 0.05. Data were analyzed by Kruskal–Wallis one-way ANOVA between lines within each NaCl treatment, since they did not meet the normality assumption. (D) Pigment degradation in salt-exposed WT and Fld-expressing plants. Seedlings were salt-treated as indicated above, and pigments were determined spectrophotometrically as described in Materials and Methods. Different letters indicate significant differences at P ≤ 0.05, according to two-way ANOVA and Holm-Sidak multiple range tests.

Two independent tobacco lines, *pfld*5-8 and *pfld*4-2, expressing similar, high amounts of Fld in chloroplasts [28], were chosen to study the effect of this flavoprotein on salt tolerance. These plants and their WT siblings were exposed to NaCl in different supports including agar and hydroponics, and several concentrations and exposure times were assayed in specimens of different age, from seedlings to 2-month old plants, to rule out that the lack of salt tolerance observed by Coba de la Peña et al. [33] in *Medicago* could be due to the experimental conditions. Representative results from a large number of experiments are shown in Fig 1 and in S1 and S2 Figs.

Twelve-day-old WT and *pfld* seedlings cultured in MS0-agar were exposed to NaCl in the same medium (Fig 1 and S1 Fig). Up to 0.1 M NaCl, only moderate effects were observed on growth and pigment integrity after 15 days of salt treatment. At 0.25 M and above, WT plants exhibited arrested development of the aerial parts (Fig 1A and 1B and S1 Fig) and severe inhibition of root elongation (Fig 1A and 1C and S1 Fig). Shoots were extensively bleached (Fig 1A and S1 Fig), reflecting significant decreases in chlorophyll and carotenoid levels (Fig 1D and S1 Fig). Expression of Fld in chloroplasts of the transgenic plants did not prevent the negative effects of NaCl on growth and leaf pigments (Fig 1 and S1 Fig).

In a different experimental set-up, 5-week-old plants grown in hydroponics became progressively wilted as they were exposed to NaCl for up to 72 h (S2A Fig). The time frame of the experiment was too short to detect effects on growth or pigment levels, but photosynthetic parameters were already affected at 0.25 M NaCl. The quantum yield of photosystem II (Φ_PSII_), which provides an estimation of electron flow through PSII, and the photochemical quenching *q*P [40], were both decreased with respect to control conditions (S2B Fig). The NPQ parameter, a measure of the extent of dissipative resources that plants need to invoke for protection of the electron transport chain from photo-oxidation, was instead increased (S2B Fig). The *Fv*/*Fm* ratio, which reflects the extent of photodamage to PSII, was not affected under these conditions (S2B Fig). Once again, neither the wilting nor the photosynthetic impairment induced by salt treatment was protected by Fld expression in the transgenic lines (S2 Fig). At 0.5 M NaCl, leaf damage was too extensive (S2A Fig) to allow measurements of photosynthetic activity. Then, our observations confirmed those obtained with *Medicago* [33], indicating that Fld-expressing plants did not display increased tolerance toward NaCl.

**Fig 2:**
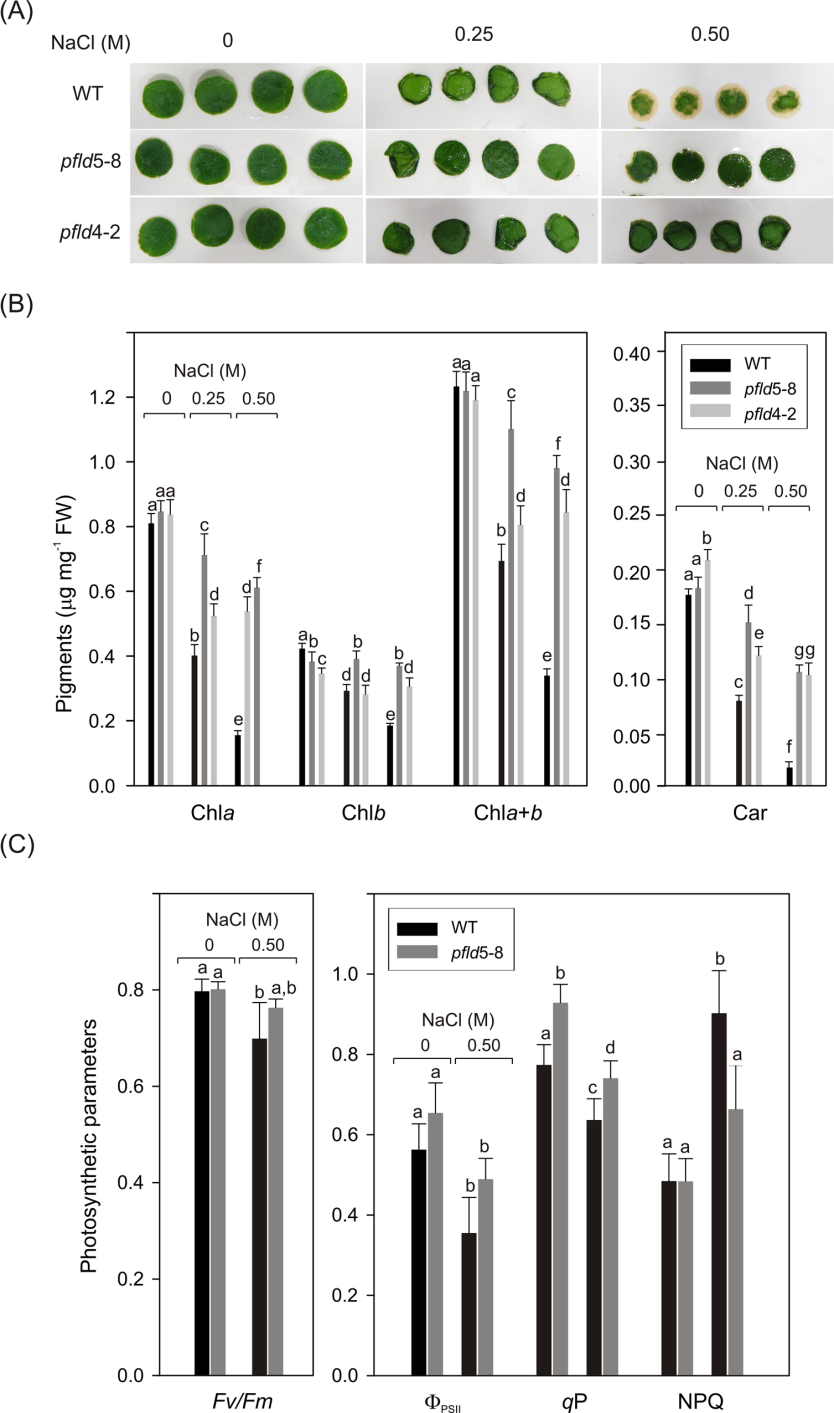
Plastid-located flavodoxin protects NaCl-exposed leaf tissue from salt toxicity. Leaf discs from 2-month-old WT and *pfld* plants grown in soil were incubated at different NaCl concentrations for various times (A). Chlorophylls (Chl) and carotenoids (Car) were determined at 72 h of treatment (B), whereas the photosynthetic parameters (C) were measured after 24 h of salt exposure. Experimental details are given in Materials and Methods. In (B) and (C), the means and standard deviations (SD) of 6 independent assays are reported. Different letters indicate significant differences at P ≤ 0.05, according to two-way ANOVA and Holm-Sidak multiple range tests.

Since salinity was reported to cause significant damage in leaves [46, 47], the salt effect was assayed directly on foliar tissue. Discs were cut from the fourth fully expanded leaves of 2-month-old plants grown in soil, and incubated with NaCl solutions of various concentrations. Under these conditions, and in contrast to the results obtained with whole plants, leaf discs from Fld-expressing lines displayed increased tolerance to salt toxicity relative to the wild type (Fig 2). Upon exposure to 0.25 M NaCl for 72 h, WT leaf tissue exhibited significant destruction of pigments, which was partially prevented in the transgenic lines (Fig 2B). At 0.5 M NaCl, Fld protection was even more pronounced (Fig 2B), with WT discs already showing extensive bleaching around the edges (Fig 2A).

Differential salt tolerance of discs from Fld-expressing plants was also evident in the photosynthetic activities. At 72 h, WT tissue was too damaged to measure PSII-associated photosynthesis, but photosynthetic differences between WT and *pfld*5-8 discs were already detectable after 24 h of treatment, in the absence of phenotypic symptoms. The *Fv*/*Fm* value exhibited a small but statistically significant decrease (~15%) in salt-stressed WT discs, while it remained unaffected in *pfld*5-8 siblings (Fig 2C). Other photosynthetic parameters (Φ_PSII_ and q*P*) were also protected in discs from *pfld*5-8 plants, whereas NPQ was significantly enhanced in WT compared to *pfld*5-8 discs (1.9-fold *vs*. 1.4-fold), indicating higher photo-oxidative stress compared to Fld-expressing leaf tissues (Fig 2C).

### Fld expression decreases LOOH accumulation in salt-stressed leaves

**Fig 3:**
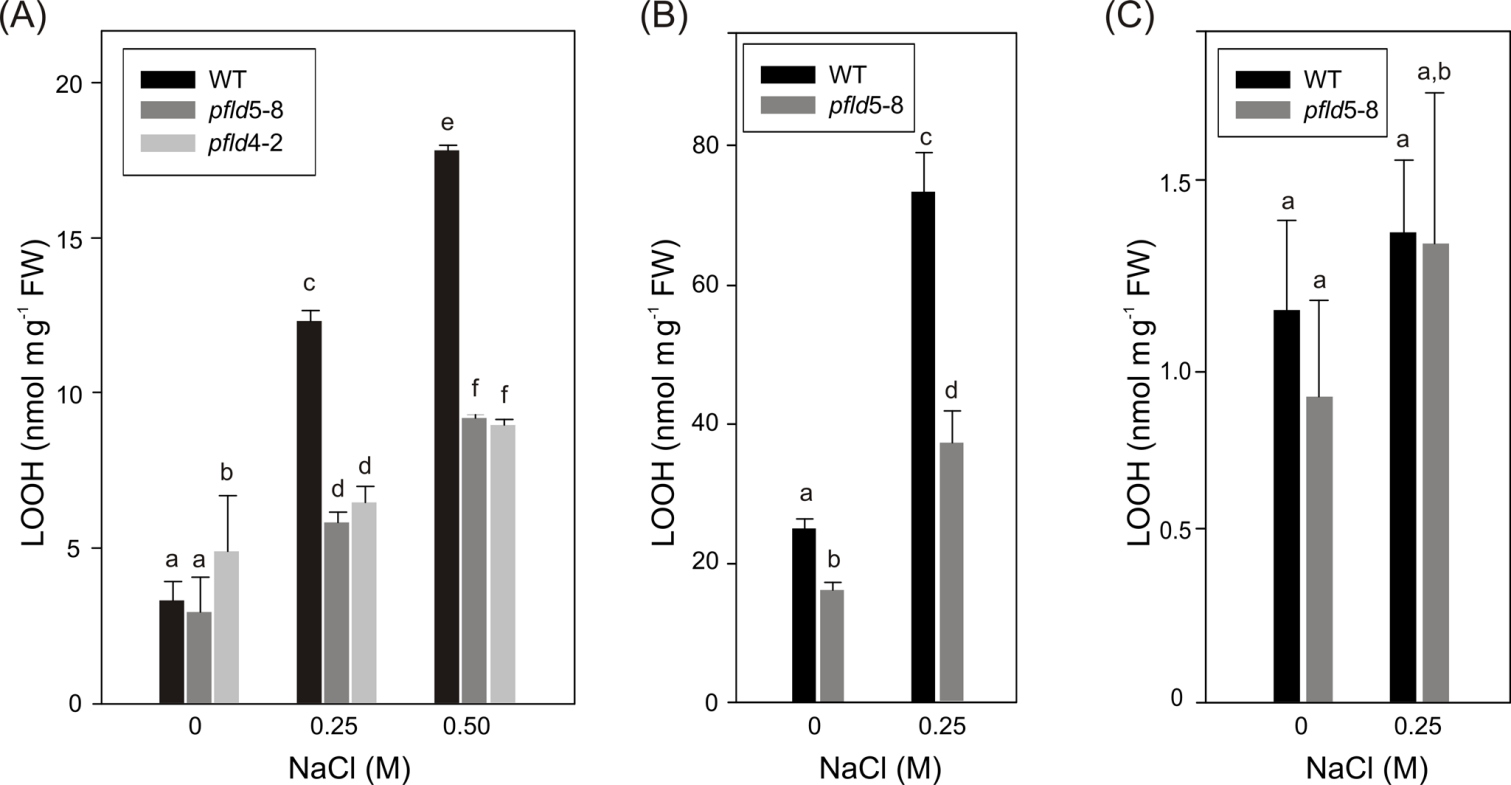
Expression of a chloroplast-targeted flavodoxin in transgenic tobacco suppresses LOOH formation in leaf tissue from salt-exposed plants and discs. Leaf discs (A) were incubated with NaCl for 72 h as described in Fig 2. Four-week-old plants were also exposed to salt for 72 h in hydroponic HM medium and LOOH determined in cleared extracts of leaves (B) and roots (C), using the xylenol orange method [41]. Means and SD values of 6 independent experiments are provided, with different letters indicating significant differences at P ≤ 0.05, according to two-way ANOVA and Holm-Sidak multiple range tests.

The amounts of LOOH present in leaves, roots and leaf discs of WT and *pfld* plants exposed to NaCl were estimated in cleared extracts by the ferrous oxidation-xylenol orange procedure [41]. Exposure of leaf discs to salinity conditions led to LOOH build-up, as previously reported [47]. At 0.25 M NaCl, the increase in LOOH levels after 72 h of incubation was ~2-fold lower in the Fld-expressing plants compared to the wild type (Fig 3A). The antioxidant protection conferred by Fld was even more significant at 0.5 M NaCl (Fig 3A). Fld expression also prevented LOOH build-up in attached leaves from plants exposed to NaCl for 72 h in hydroponic medium (Fig 3B).

To visualize ROS cellular localization, leaves from salt-treated plants were collected at 72 h of incubation and stained with the fluorescent probe DCFDA for inspection by confocal microscopy. As expected, most of the label was recovered in chloroplasts of illuminated WT leaves, co-localizing with chlorophyll auto-fluorescence (S3 Fig). Image analysis indicates that ROS levels in *pfld* leaves were ~47% of those obtained in the wild type (S3 Fig). Then, Fld expression specifically decreased ROS build-up in chloroplasts of plants exposed to salinity stress.

Finally, LOOH accumulation in roots from the same salt-treated plants was very low compared to leaves of the same salt-treated plants, was not significantly different from siblings incubated in the absence of NaCl, and was not affected by Fld expression (Fig 3C), suggesting that oxidative stress did not play a major role in root cell damage during salt exposure.

### Salt affected root integrity and function to a similar extent in WT and Fld-expressing lines

Results described in the preceding sections indicate that chloroplast-targeted Fld was able to increase tolerance against NaCl only when the salt was directly applied to the leaf tissue. On the other hand, the lack of effect on whole plants, despite significant ROS decrease in attached leaves (Fig 3B), suggests that salt-sensitive targets which are not protected by Fld expression do exist outside the leaf. When plants are exposed to NaCl in hydroponics, agar or soil, the salt enters the root and travels through the xylem [48] before reaching the leaf. The root is therefore a primary candidate to contain such putative targets.

**Fig 4:**
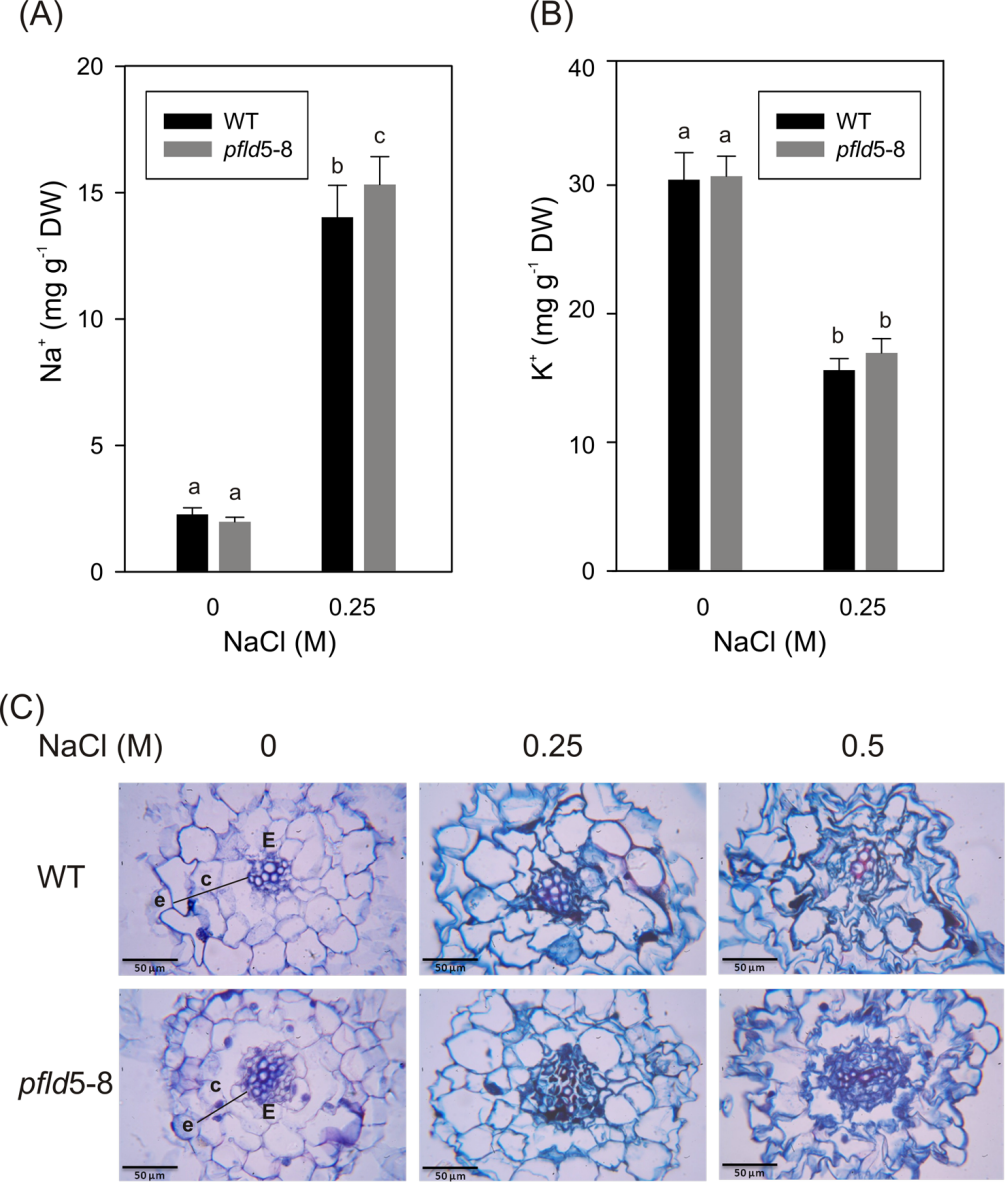
Ion contents and tissue damage in roots of salt-treated plants. Four-week-old plants grown in HM hydroponics were incubated with NaCl for 72 h, and Na^+^ (A) and K^+^ (B) contents were determined in root extracts as described in Materials and Methods. Experiments were carried out in quadruplicate. Different letters indicate significant differences at P ≤ 0.05, according to two-way ANOVA and Holm-Sidak multiple range tests. (C) Structural analysis of root tissue damage. Cross-sections of the elongation zone in roots from 27-day-old WT and *pfld* seedlings incubated with NaCl in MS0-agar for 15 days were prepared as described in Materials and Methods and visualized at the light microscope. c: cortex, e: epidermis, E: endoderm.

As shown in Fig 1, root elongation was severely inhibited in plants exposed to NaCl. The degree of inhibition in Fld-expressing lines was similar to that of the wild type (Fig 1C). Moreover, the presence of the flavoprotein in chloroplasts from the *pfld*5-8 line had only marginal effects on Na^+^ uptake and accumulation into root tissue under salt stress. When 4-week-old plants were exposed to 0.25 M NaCl added to the hydroponic solution for 72 h, root Na^+^ contents increased about 7-fold relative to untreated plants, irrespective of the presence of Fld (Fig 4A), whereas root K^+^ levels showed a moderate decline (Fig 4B), in agreement with previous reports [49–51].

Na^+^ contents in leaves of salt-treated WT and *pfld*5-8 plants increased about 12-fold (S4 Fig) compared to untreated siblings, whereas direct NaCl application to leaf discs led to much higher accumulation of the cation in the tissue, amounting to an increase of ~140-fold relative to discs incubated in water for the same time period (S4 Fig). Potassium contents decreased in leaf discs but not in attached leaves (S4 Fig).

The effects of NaCl on root integrity were visualized at the cellular level by light microscopy. Transverse sections of the elongation zone in roots from untreated WT and transgenic plants revealed no major anatomical differences between lines (Fig 4C). Salt stress caused extensive disorganization and damage of the respective tissues, with numerous amoeboid protrusions and folds in epidermal (e) and cortical (c) cells at 0.5 M NaCl (Fig 4C). Swelling was only marginal. Fld expression failed to prevent salt-dependent damage in *pfld* roots (Fig 4C).

**Fig 5:**
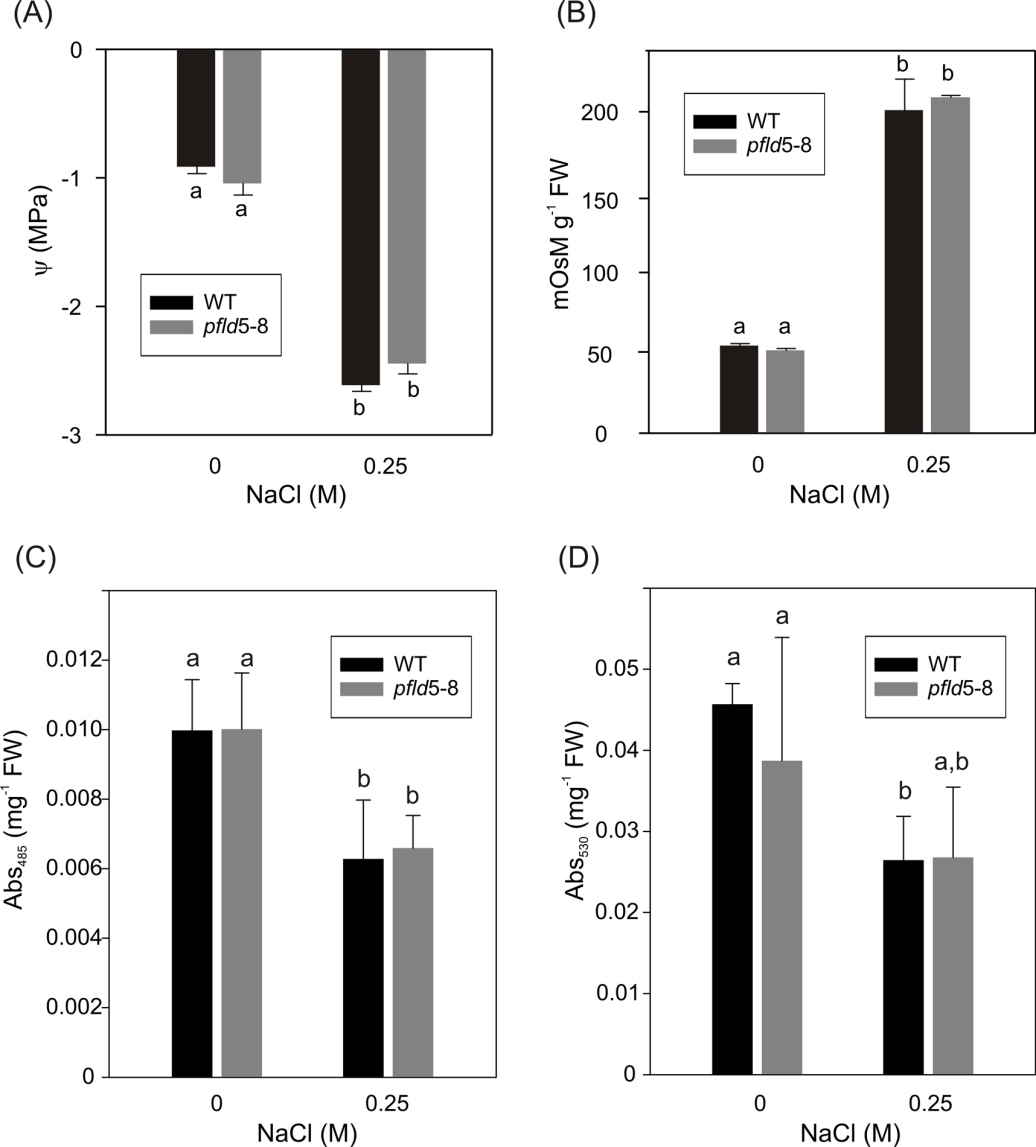
Chloroplast flavodoxin does not prevent the decline of osmotic pressure and viability in salt-exposed tobacco roots. Twelve-day-old seedlings were incubated for 15 days with 0.25 M NaCl in MS0-agar plates and the osmotic potential (ψ) of excised whole roots was determined as described in Materials and Methods (A). The osmolarity of replicate samples was also measured in cleared extracts (B) using a freezing point osmometer. Each value is the mean of 6 replicates plus SD. For the evaluation of root and leaf viabilities, 12-day-old seedlings were salt-treated as in panels A and B, except that incubation was limited to 11 days. Metabolic activities were determined in cleared leaf (C) and root (D) extracts by the TTC reduction procedure [45]. Means and SD values were determined by averaging 6 independent experiments. Different letters indicate significant differences at P ≤ 0.05, according to two-way ANOVA and Holm-Sidak multiple range tests.

The osmotic pressure of salt-treated roots was monitored in WT and Fld-expressing plants by two complementary procedures: determination of the osmotic potential in whole root tissue (Fig 5A), and estimation of the osmolarity in total root extracts (Fig 5B). Both methods yielded similar results: an increase of the osmotic pressure upon salt exposure, with no significant differences between lines (Fig 5A and 5B).

The osmotic component of salt stress can be mimicked by the use of nonionic osmolytes such as PEG, therefore uncoupling osmotic stress from possible ionic or nutritional effects. The osmotic pressure of the NaCl concentrations we used in the experiments described here varied from -0.22 to -2.22 MPa (S1 Fig), with significant salt effects becoming obvious above 0.25 M NaCl (~-1.1 MPa). We therefore chose an intermediate value between the two highest NaCl concentrations. Exposure of 15-day-old tobacco seedlings grown in MS0-agar plates to a PEG 8000 concentration corresponding to ~-1.5 MPa for 11 additional days resulted in almost complete inhibition of both shoot and root growth. Once again, expression of Fld failed to improve the osmotic tolerance of the transformants relative to the corresponding wild type (S5 Fig).

Finally, root and leaf viabilities were compared by measuring their ability to reduce the broad-range dehydrogenase substrate TTC, a widely employed biochemical marker of metabolic activity [47, 52]. The TTC reduction activity was higher in leaves (Fig 5C) than in roots (Fig 5D), and declined to 55 and 60%, respectively, when plants grown in MS0-agar plates for 12 days were exposed to 0.25 M NaCl in the same medium for additional 11 days (Fig 5C and 5D). The loss of metabolic viability was similar in WT and Fld-expressing plants (Fig 5C and 5D), indicating that salt-dependent enzyme inhibition was not prevented by Fld expression.

## Discussion

Expression of a plastid-directed Fld in higher plants decreased ROS build-up during episodes of environmental stress, correlating with enhanced tolerance to various stresses of agronomical relevance such as drought, iron limitation, extreme temperatures and excess irradiation [28–30]. The protective effect of the flavoprotein could be assessed at the whole plant level by improved growth and survival, and lower degree of wilting and bleaching [28–30]. On the other hand, augmented production and accumulation of ROS as a consequence of salt exposure have been extensively documented for many plant species [7, 12, 14, 53], suggesting that Fld could also enhance salinity tolerance. Results obtained with tobacco plants exposed to NaCl under various conditions indicated otherwise: Fld-expressing lines showed the same degree of growth arrest, pigment degradation and photosynthesis impairment as their WT siblings (Fig 1, S1 and S2 Figs). These results concur with a previous observation in *Medicago* plants [33]. Noteworthy, when the NaCl treatment was directly applied on leaf tissue, the presence of Fld in chloroplasts increased salt tolerance (Fig 2), indicating that plastid-generated ROS do contribute to the damage experienced by photosynthetic cells. These results might explain why Fld is able to improve salt tolerance of cyanobacterial cells, as has been extensively documented [34, 35, 54]. In the case of plants, in which salt initially challenges the root cells and reaches the leaf via the vascular system, it is likely that root damage is the main source of growth inhibition.

Under the conditions employed in the present research, salt-dependent ROS accumulation in roots was negligible and not affected by the presence of Fld (Fig 3C). Indeed, Fld expression failed to protect plants against the effect of NaCl on root cell structure (Fig 4C), osmolarity (Fig 5A and 5B), viability (Fig 5C and 5D), and Na^+^ uptake (Fig 4A and S4 Fig). The decline in root tissue osmotic pressure (Fig 5A and 5B) is most likely the cause for the lack of differences in growth between WT and Fld-expressing lines (Fig 1 and S1 Fig).

Salt-treated whole plants expressing Fld in chloroplasts displayed lower levels of ROS accumulation in leaves (Fig 3B), but this was not accompanied by better growth (Fig 1A–1C and S1 Fig), pigment integrity (Fig 1D), photosynthetic activity (S2 Fig), or leaf viability (Fig 5C). This outcome contrasts with the increased tolerance exhibited by the same transformants against other sources of environmental stress such as extreme temperatures, water and iron deficit, excess irradiation and pathogens [28–30, 55].

As indicated before, ROS can be synthesized in various sub-cellular compartments, but it is not clear how and which of these ROS sources contribute more to plant damage and response(s) during salt stress. ROS propagation and oxidative damage caused by NaCl exposure were shown to be prominent in leaves of maize and cowpea, but not in roots [46, 47]. Those reports agree with our observations on the lack of ROS build-up in roots from salt-treated WT and *pfld* plants (Fig 3C). Also, induction of different members of the antioxidative response has been observed in leaves of various plant species exposed to salinity, including pea [56], olive [57], cowpea [46], maize [47], and mangrove [58]. Attempts to correlate induction of these enzymes with tolerance to NaCl stress in salt-sensitive and salt-tolerant cultivars had limited success [12, 59]. An alternative, complementary approach to identify the source(s) of ROS during salt stress and to evaluate their effects on plant tolerance is to increase the contents of well characterized components of the antioxidative response. For instance, overexpression of different glutathione *S*-transferase (GST) isoforms in tobacco and Arabidopsis led to decreased ROS accumulation and lower levels of lipid peroxidation in plants exposed to salinity [60, 61].

With respect to the specific contribution of chloroplast ROS during salt stress, down-regulation of ROS build-up in plastids by overexpression of several antioxidant enzymes increased NaCl tolerance in cotton [62] and tobacco [58]. Moreover, Wu et al. [63] have recently shown that plastid-generated ROS play a significant role in NaCl-dependent K^+^ efflux from wheat leaf mesophyll, a decline that would contribute to the nutritional deficit component of salt toxicity. These results suggested that ROS accumulation in chloroplasts was a central component of plant damage during salt exposure and that manipulation of chloroplast scavenging enzymes might help to design salt-tolerant crops. Other reports, however, challenged these expectations. The lack of a thylakoid-associated ascorbate peroxidase (APX) in an Arabidopsis mutant, which was expected to increase salt sensitivity, resulted instead in increased tolerance, whereas knockout plants deficient in the major cytosolic APX grew better than WT siblings under hyperosmotic or saline conditions [64, 65].

In conclusion, our results indicate that salt stress causes oxidative damage in leaves and a cellular osmotic imbalance in roots, with loss of metabolic viability in both tissues. Lowering of ROS levels by Fld expression may be advantageous for leaf cell survival under stress conditions, as observed in NaCl-treated leaf discs, but is not enough to achieve a recovery of leaf growth and root elongation. A similar situation was observed in transgenic Arabidopsis lines expressing GST, which failed to recover from salt-dependent growth arrest despite significant inhibition of ROS build-up [61]. Interestingly, ROS-related transcripts encoding transcription factors and signaling proteins displayed contrasting up-regulation patterns in response to stress depending on the tissue: induction under osmotic stress conditions occurred almost exclusively in shoots, while it was limited to roots during salinity [7]. These observations suggest that global ROS production and scavenging might be of little importance for salt tolerance, while precise ROS contributions, finely regulated in time and space, might play a greater role in this process.

Given the growing concern about salinization at a planetary scale, largely due to faulty irrigation practices [1, 39, 66], development of salt-tolerant crops is a major goal of plant biotechnology to complement the adoption of more rational agronomical strategies. Although most attempts to improve salt tolerance have so far focused on engineering ion transport devices (reviewed in [5, 67]), the oxidative component of salt stress has also been targeted for transgenic intervention. Our results raise a cautionary note on these attempts, and underscore the need for a better understanding of ROS production and detoxification in defined compartments of the plant tissue in order to manipulate these pathways for better salt stress endurance.

## Supporting Information

S1 Fig Flavodoxin expression has no effect on the tolerance of tobacco plants against salinity. Twelve-day old WT and *pfld* seedlings cultured in MS0-agar plates were incubated with NaCl in the same medium and photographed after 15 days of treatment. Osmotic pressures (MPa) resulted from the salt added to the culture medium were calculated using van’t Hoff equation with a correction coefficient of 1.8 [38].
(TIF)

S2 Fig Chloroplast flavodoxin does not prevent inhibition of photosynthetic activities after short-term exposure of tobacco plants to NaCl in hydroponia. Five-week-old plants grown in hydroponics were incubated with NaCl in the same HM medium, and pictures were taken at 72 h (A). Photosynthetic parameters (B) were determined in leaves from plants treated with 0.25 M NaCl. Means and SD values are indicated. Different letters show significant differences at P ≤ 0.05 according to two-way ANOVA and Holm-Sidak multiple range tests.
(TIF)

S3 Fig Flavodoxin prevents ROS accumulation in chloroplasts of salt-treated transgenic tobacco plants. Leaves from plants incubated in hydroponic HM with or without 0.25 M NaCl for 72 h were loaded with 50 μM DCFDA for ROS detection as described in Materials and Methods. Images were recorded with an Eclipse TE– 2000 –E2 Nikon confocal microscope (excitation at 488 nm, emission at 515/530 nm). From left to right, DCFDA fluorescence (green), chlorophyll (Chl) autofluorescence (red) and merge. Numerals on the extreme right show green fluorescence intensities in arbitrary units (A. U.) as estimated in 4 replicate samples using the Image J software. Scale bar = 20 μm.
(TIF)

S4 Fig Analysis of ion contents in leaf tissue of plants and discs subjected to salt treatment. Four-week-old plants grown in hydroponics were incubated with NaCl for 72 h and Na^+^ (A) and K^+^ (C) levels were determined in leaf extracts as described in Materials and Methods. Discs were salt-treated for the same time period (B, D), as described in the legend to Fig 2. Bars in the inset of panel B show Na^+^ levels of untreated discs in a different scale. Experiments were carried out in quadruplicate, and means and SD bars are shown in the figure. Asterisks indicate significant differences of the means between lines at P ≤ 0.05, according to Kruskal–Wallis one-way ANOVA and Tukey multiple range tests.
(TIF)

S5 Fig Osmotic effects of PEG 8000 on wild-type and flavodoxin-expressing plants. Fifteen-day-old seedlings were incubated with PEG-8000 in MS0-agar plates, and photographed after 11 days of treatment. The concentration of PEG 8000 used corresponds to an osmotic pressure of ~-1.5 MPa.
(TIF)
