# Supplementary figures and images for: Suppression of Reactive Oxygen Species Accumulation in Chloroplasts Prevents Leaf Damage but Not Growth Arrest in Salt-Stressed Tobacco Plants
Source: PLoS One. 2016 Jul 21;11(7):e0159588. doi: 10.1371/journal.pone.0159588 (PMC4956149; doi:10.1371/journal.pone.0159588)

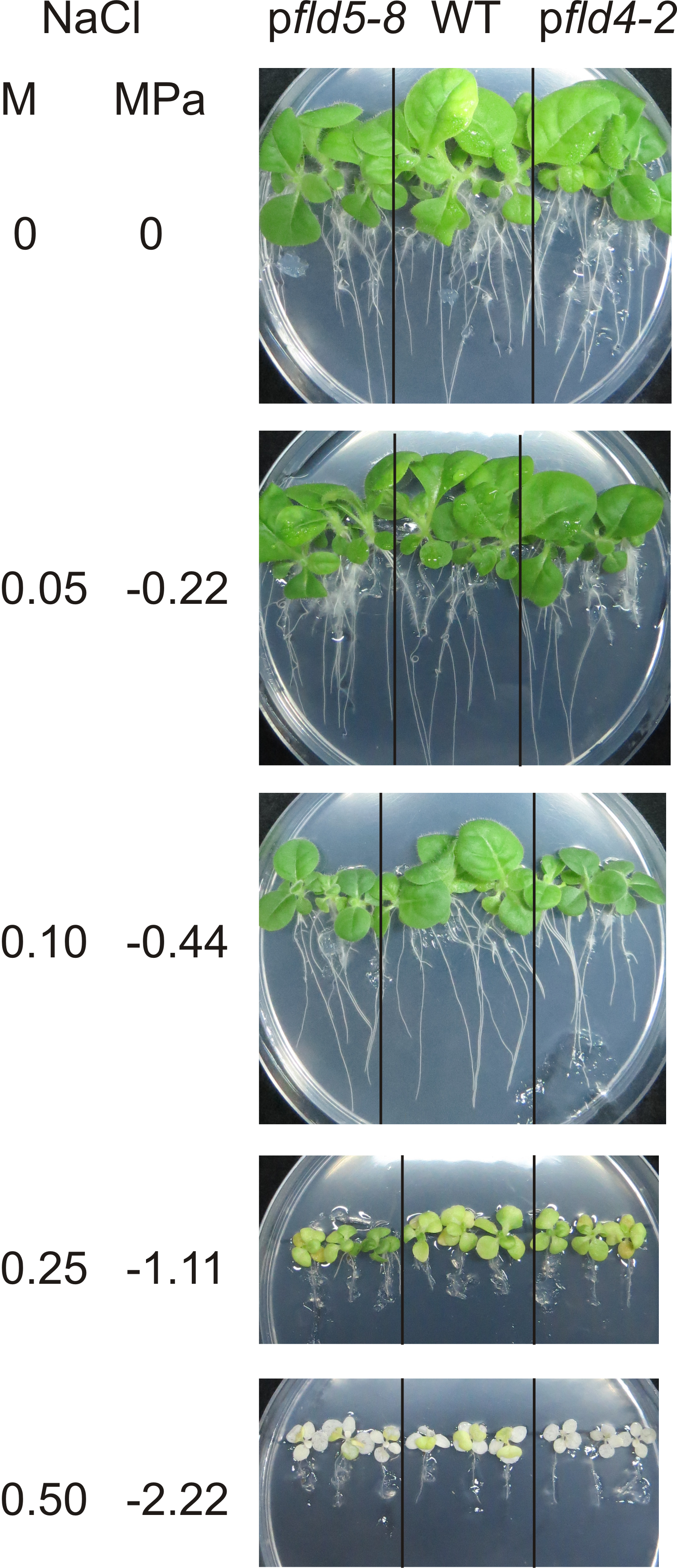

Supplement: S1 Fig — Twelve-day old WT and pfld seedlings cultured in MS0-agar plates were incubated with NaCl in the same medium and photographed after 15 days of treatment. Osmotic pressures (MPa) resulted from the salt added to the culture medium were calculated using van’t Hoff equation with a correction coefficient of 1.8 [38]. (TIF) [file pone.0159588.s001.TIF]

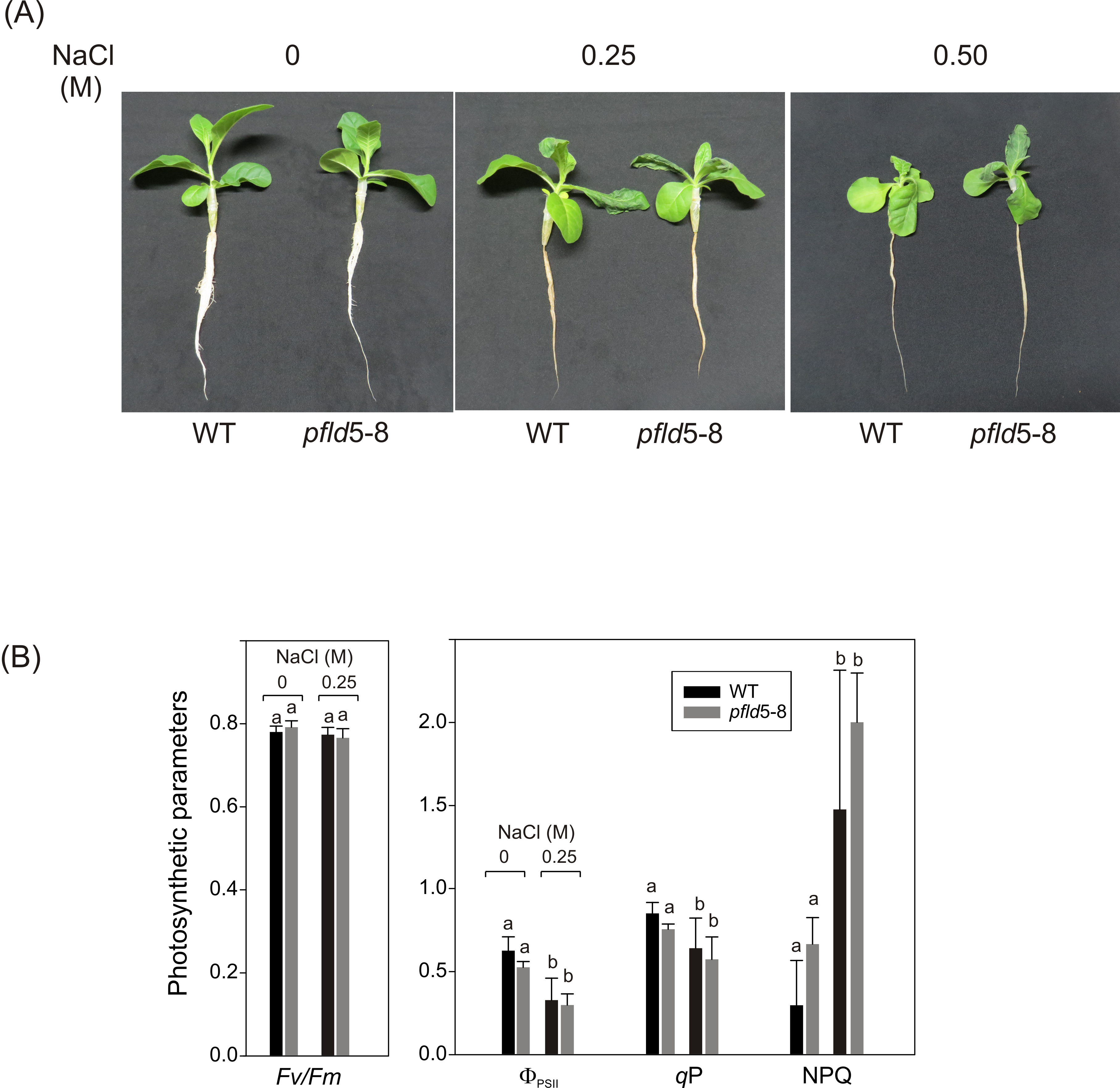

Supplement: S2 Fig — Five-week-old plants grown in hydroponics were incubated with NaCl in the same HM medium, and pictures were taken at 72 h (A). Photosynthetic parameters (B) were determined in leaves from plants treated with 0.25 M NaCl. Means and SD values are indicated. Different letters show significant differences at P ≤ 0.05 according to two-way ANOVA and Holm-Sidak multiple range tests. (TIF) [file pone.0159588.s002.TIF]

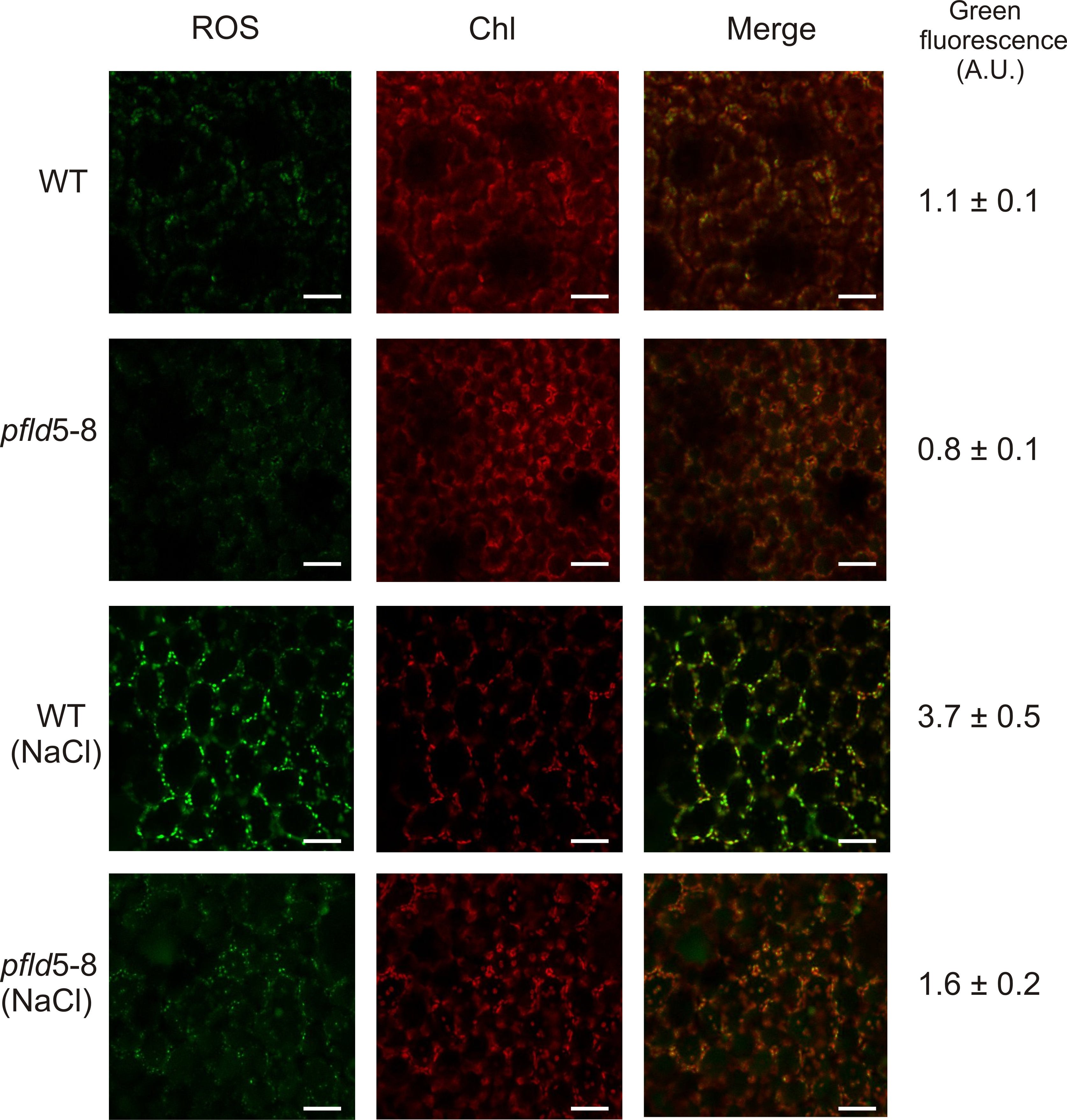

Supplement: S3 Fig — Leaves from plants incubated in hydroponic HM with or without 0.25 M NaCl for 72 h were loaded with 50 μM DCFDA for ROS detection as described in Materials and Methods. Images were recorded with an Eclipse TE– 2000 –E2 Nikon confocal microscope (excitation at 488 nm, emission at 515/530 nm). From left to right, DCFDA fluorescence (green), chlorophyll (Chl) autofluorescence (red) and merge. Numerals on the extreme right show green fluorescence intensities in arbitrary units (A. U.) as estimated in 4 replicate samples using the Image J software. Scale bar = 20 μm. (TIF) [file pone.0159588.s003.TIF]

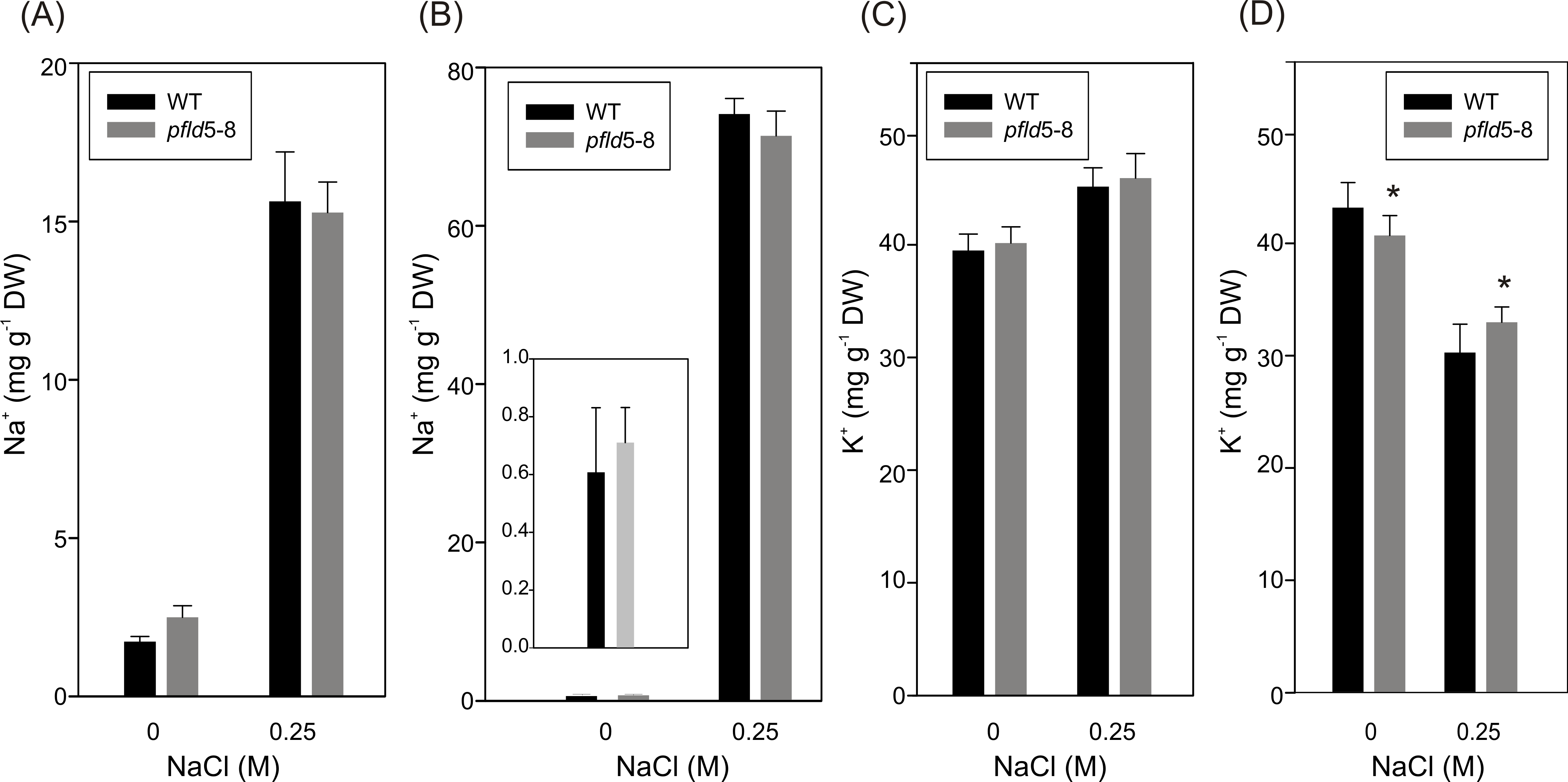

Supplement: S4 Fig — Four-week-old plants grown in hydroponics were incubated with NaCl for 72 h and Na+ (A) and K+ (C) levels were determined in leaf extracts as described in Materials and Methods. Discs were salt-treated for the same time period (B, D), as described in the legend to Fig 2. Bars in the inset of panel B show Na+ levels of untreated discs in a different scale. Experiments were carried out in quadruplicate, and means and SD bars are shown in the figure. Asterisks indicate significant differences of the means between lines at P ≤ 0.05, according to Kruskal–Wallis one-way ANOVA and Tukey multiple range tests. (TIF) [file pone.0159588.s004.TIF]

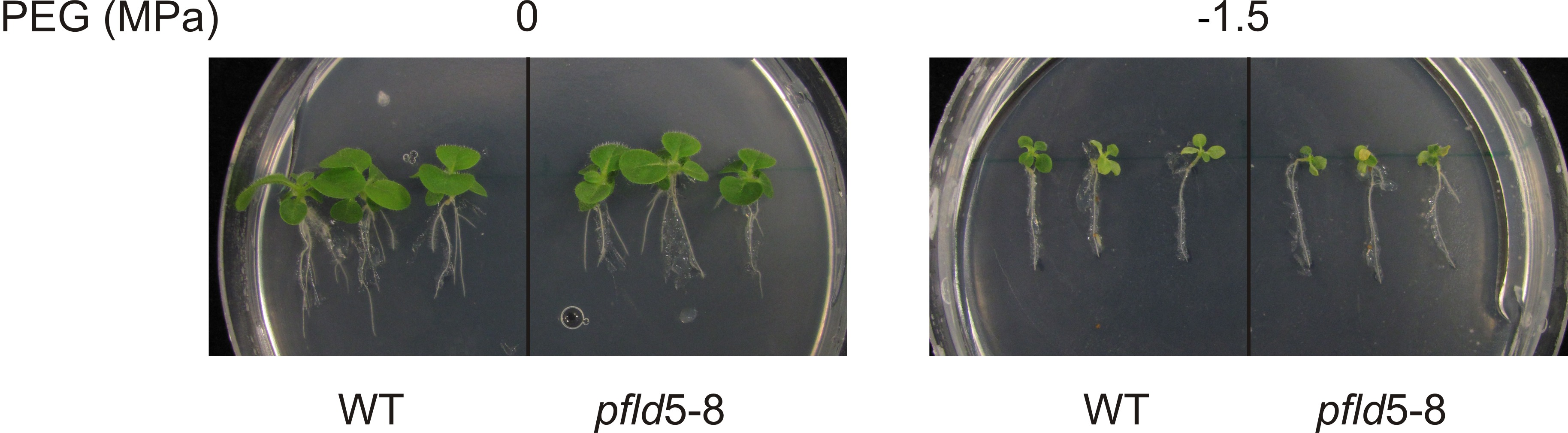

Supplement: S5 Fig — Fifteen-day-old seedlings were incubated with PEG-8000 in MS0-agar plates, and photographed after 11 days of treatment. The concentration of PEG 8000 used corresponds to an osmotic pressure of ~-1.5 MPa. (TIF) [file pone.0159588.s005.tif]
